# Read-Out of Dynamic Morphogen Gradients on Growing Domains

**DOI:** 10.1371/journal.pone.0143226

**Published:** 2015-11-24

**Authors:** Patrick Fried, Dagmar Iber

**Affiliations:** 1 Department of Biosystems Science and Engineering, ETH Zürich, Basel, Switzerland; 2 SIB Swiss Institute of Bioinformatics, Basel, Switzerland; University of Dayton, UNITED STATES

## Abstract

Quantitative data from the *Drosophila* wing imaginal disc reveals that the amplitude of the Decapentaplegic (Dpp) morphogen gradient increases continuously. It is an open question how cells can determine their relative position within a domain based on a continuously increasing gradient. Here we show that pre-steady state diffusion-based dispersal of morphogens results in a zone within the growing domain where the concentration remains constant over the patterning period. The position of the zone that is predicted based on quantitative data for the Dpp morphogen corresponds to where the Dpp-dependent gene expression boundaries of *spalt* (*sal*) and *daughters against dpp* (*dad*) emerge. The model also suggests that genes that are scaling and are expressed at lateral positions are either under the control of a different read-out mechanism or under the control of a different morphogen. The patterning mechanism explains the extraordinary robustness that is observed for variations in Dpp production, and offers an explanation for the dual role of Dpp in controlling patterning and growth. Pre-steady-state dynamics are pervasive in morphogen-controlled systems, thus making this a probable general mechanism for the scaled read-out of morphogen gradients in growing developmental systems.

## Introduction

**Fig 1:**
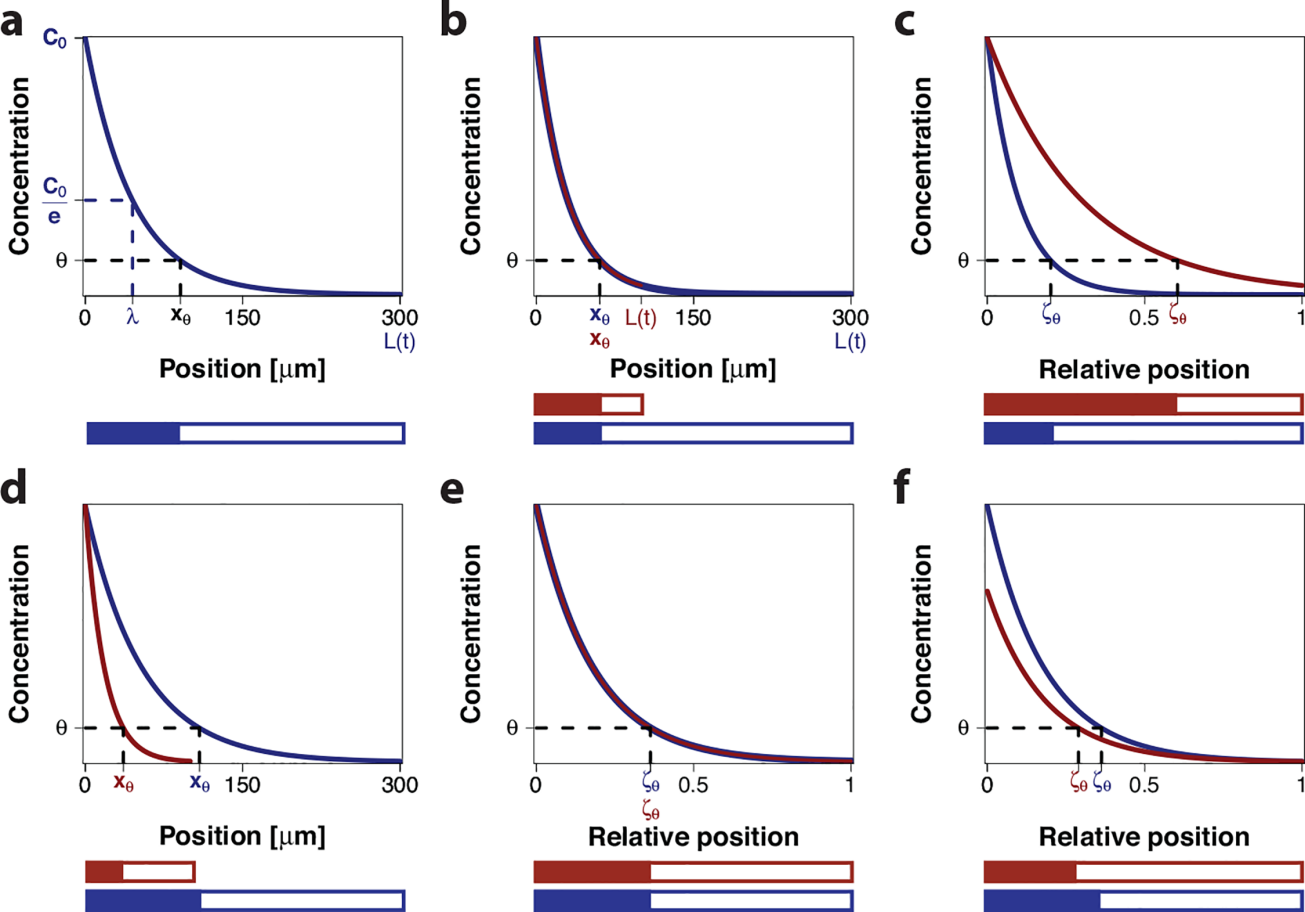
Morphogen gradients. (a) An exponential morphogen gradient (Eq (1)) with amplitude *c*_0_ on a domain with length *L* = 300 μm. The position where the concentration equals $\frac{c_{0}}{e}$, the characteristic length scale, is denoted *λ*. According to the French Flag model, a constant concentration threshold *θ* defines the boundary position *x*_*θ*_ between two subdomains, shown below in blue and white. (b) Two gradients with the same length scale *λ* and the same amplitude *c*_0_ on differently sized domains have a common boundary position, *x*_*θ*_. (c) On a rescaled domain the gradients in panel b define different relative boundary positions, *ζ*_*θ*_, leading to different relative lengths of the subdomains, shown below in red and blue. (d) Two gradients with a common ratio $\frac{\lambda }{L}$ and the same amplitude *c*_0_ on differently sized domains have different absolute read-out positions, *x*_*θ*_. (e) On a rescaled domain, the gradients from panel d overlap and thus define a common relative boundary position *ζ*_*θ*_. Therefore, the relative lengths of the subdomains are the same, shown below in red and blue. (f) For different amplitudes *c*_0_ for the scaled gradients in panels d,e, the relative boundary positions are no longer the same, and the relative lengths of the subdomains thus differ, shown below in red and blue.

Morphogens control the emergence of spatial patterns during embryonic development [1–5]. According to the French Flag model, a morphogen gradient emerges across the field of cells and cells sense the local morphogen concentration and assume a fate according to whether the sensed concentration is above or below a given concentration threshold [6]. While the exact shape of morphogen gradients has been a matter of debate [7,8], quantitative experimental data for the morphogen Decapentaplegic (Dpp) in the *Drosophila* wing imaginal disc show that the Dpp morphogen forms a gradient across its patterning domain that can be fitted well by an exponential function of the form

$$c(x)=c_{0}\cdot \exp(-x/\lambda ),$$ (1)

where *c*_0_, the amplitude of the gradient, corresponds to the concentration at the source and *λ* is the characteristic length of the exponential gradient (Fig 1A) [9,10]. Dpp defines the expression boundaries of several genes, including those of *spalt* (*sal*) and *daughters against dpp* (*dad*) [11,12]. According to the French Flag Model, the boundary position, *x*_*θ*_, of a gene expression domain (Fig 1A) is then defined by a concentration threshold *c*(*x*) = *θ* and thus by

$$x_{\theta }=\lambda \cdot \log\left(\frac{c_{0}}{\theta }\right).$$ (2)

The read-out problem is further complicated by the fact that the patterns in the *Drosophila* wing disc emerge as the domain is growing out. If the morphogen formed a stable, steady-state gradient with a constant read-out position (Fig 1B) then the relative position would move closer to the source as the domain is growing out (Fig 1C). Intriguingly, it has been found that the Dpp gradient widens on the growing domain (Fig 1D) in a way that the gradient length increases in parallel to the increase in the wing disc length, a phenomenon that is referred to as scaling [10] (Fig 1E). Since the wing disc domain grows largely uniformly [10] and the apical surface area of cells only shrinks at later stages in parts of the wing disc [10,13–15], cells roughly maintain their relative positions over time. As a result, the relative position of the cells with respect to the gradient remains constant.

A number of different models have been proposed to explain the scaling of morphogen gradients [16–20]. Most scaling mechanisms assumed the morphogen gradient to be in steady-state. The quantitative data of the Decapentaplegic (Dpp) gradient in the *Drosophila* wing imaginal disc, however, show that the morphogen gradient is highly dynamic and that the gradient increases continuously over time [10]. A pre-steady state diffusion-based transport model is indeed more consistent with the quantitative data and explains the observed scaling behavior [20]. Thus, as a result of the diffusion-based pre-steady state dispersal, the length of the exponential gradient increases proportional to the square root of time, as indeed observed in the data [10]. In conclusion, the scaling of the Dpp gradient in the wing disc is not perfect. Nonetheless, in particular, at later stages the gradient expansion is almost linearly proportional to the domain expansion [10].

While the pre-steady state dispersal explains the scaling behaviour, the question of how a continuously increasing and scaling gradient can be read out to result in scaling target genes has remained unanswered. Scaling alone cannot explain how the Dpp morphogen gradient can define expression boundaries on the growing domain because the Dpp gradient amplitude increases continuously with time [10] (Fig 1F). Accordingly, cells would be expected to see different Dpp concentrations over time such that the threshold-based relative read-out positions,

$$\zeta_{\theta }(t)=\frac{x_{\theta }}{L(t)}=\frac{\lambda }{L(t)}\log\left(\frac{c_{0}}{\theta }\right),$$ (3)

would be shifted out over time (Fig 1F). Quantitative experimental data, however, confirm that also the boundaries of the Dpp-controlled gene expression domains scale with the growing domain [21]. A mechanism must thus be in place that permits a constant relative read-out position, *ζ*_*θ*_(*t*), over time in spite of an increasing concentration gradient. It is an open question how a continuously increasing gradient could be read out [22].

We now show that the imperfect scaling of the pre-steady state gradient also resolves the conundrum of how expression boundaries can be robustly defined by an increasing gradient on a growing domain. We show that as a result of imperfect scaling, a zone exists on the domain where the Dpp concentration remains almost constant over time. Using published experimental data together with our data-based model of the Dpp gradient in the *Drosophila* wing imaginal disc, we confirm that predicted location of this zone coincides with the position where the Dpp-dependent gene expression boundaries of *sal* and *dad* emerge. The scaling of more lateral gene expression boundaries cannot be explained by this mechanism. Intriguingly, the scaled lateral gene expression domains that are affected by Dpp, i.e. brinker (brk) and optomotor blind (omb), are mainly controlled by another morphogen, Glass bottom boat (Gbb) [23]. Finally, we show that the dual role of Dpp in controlling both growth and patterning increases the robustness of the patterning mechanism to variations in Dpp production. Pre-steady-state dynamics are pervasive in morphogen-controlled systems, thus making this a probable general mechanism for the scaled read-out of morphogen gradients in growing developmental systems.

## Results

### Read-out of a morphogen gradient on a growing domain

**Fig 2:**
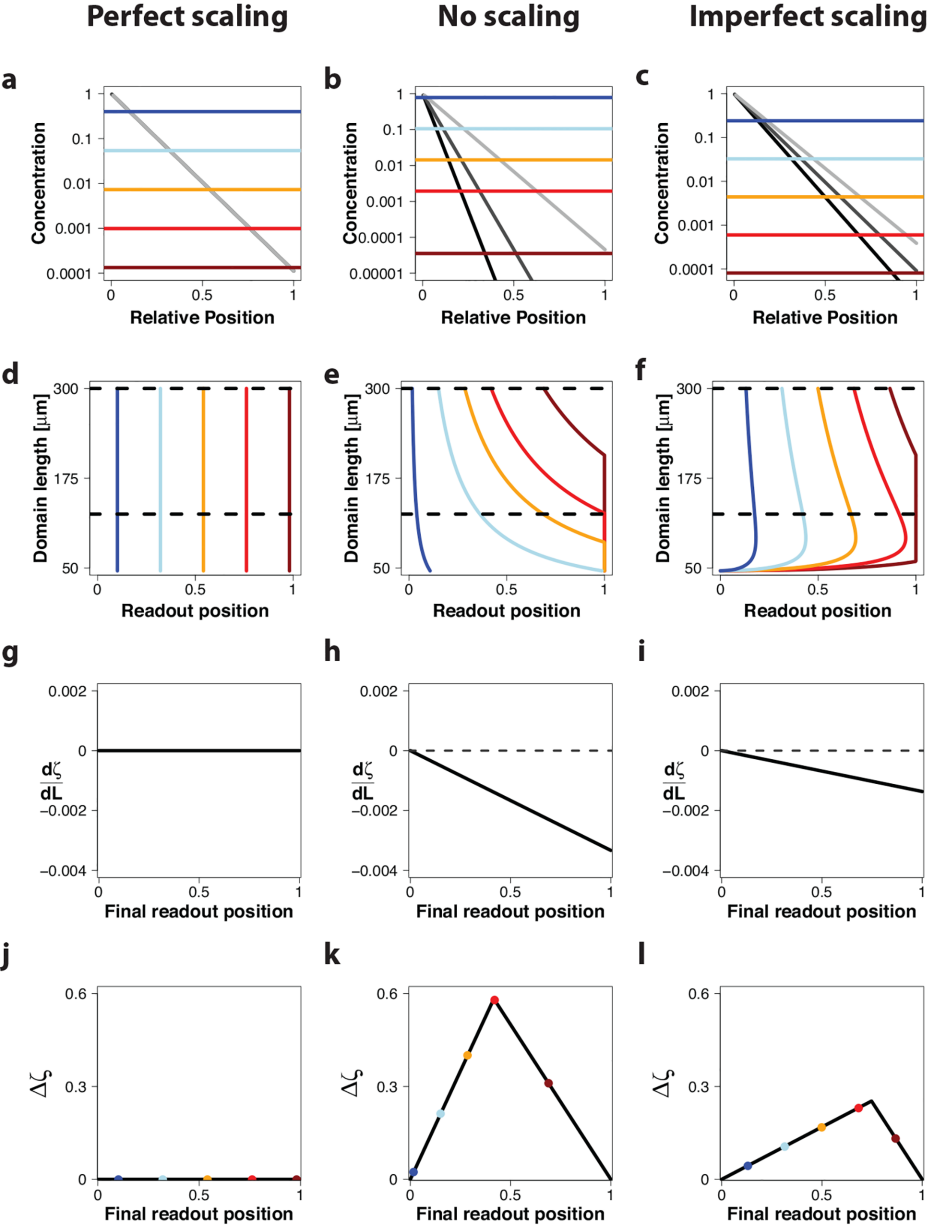
Read-out of morphogen gradients with constant amplitude *c*_0_ on a growing domain. Gradient read-out for a gradient with constant amplitude, *c*_0_, in case of (left column) perfect scaling with *λ* = *k*_1_ ⋅ *L*(*t*); *k*_1_ = 0.11 (ref [10]), (center column) absence of scaling with *λ* = 20.2 *μm* (ref [9]), and (right column) imperfect scaling with $\lambda =\sqrt{\frac{D}{2v_{g}}L(t)-\frac{D}{2v_{g}}L_{0}}$ with $\frac{D}{2v_{g}}=3\;\mu m$ and *L*_0_ = 46 *μm* (ref [20]). (a-c) A morphogen gradient at three timepoints (*L*(*t*) = 100 μm (light grey), *L*(*t*) = 200 μm (dark grey), *L*(*t*) = 300 μm (black)) on a domain that is scaled with respect to the current length of the domain, *L*(*t*). Five different concentration thresholds are shown as differently colored lines. (d-f) Trajectories of the positions, *ζ*_*θ*_, where the different threshold concentration, shown in panels a-c, are attained. The dashed, black horizontal lines mark the time interval that is further analysed in panels j-l. (g-i) The derivative of the relative read-out position with respect to the domain length, $\frac{d\zeta_{\theta }}{dL}$, evaluated at different final read-out positions. (j-l) The maximal deviation of the relative read-out positions, Δ*ζ*, in the interval from 125 μm to 300 μm (marked by dashed, black horizontal lines in panels d-f) for different final read-out positions. The colored dots indicate the final read-out position of the thresholds shown in panels a-c.

**Fig 3:**
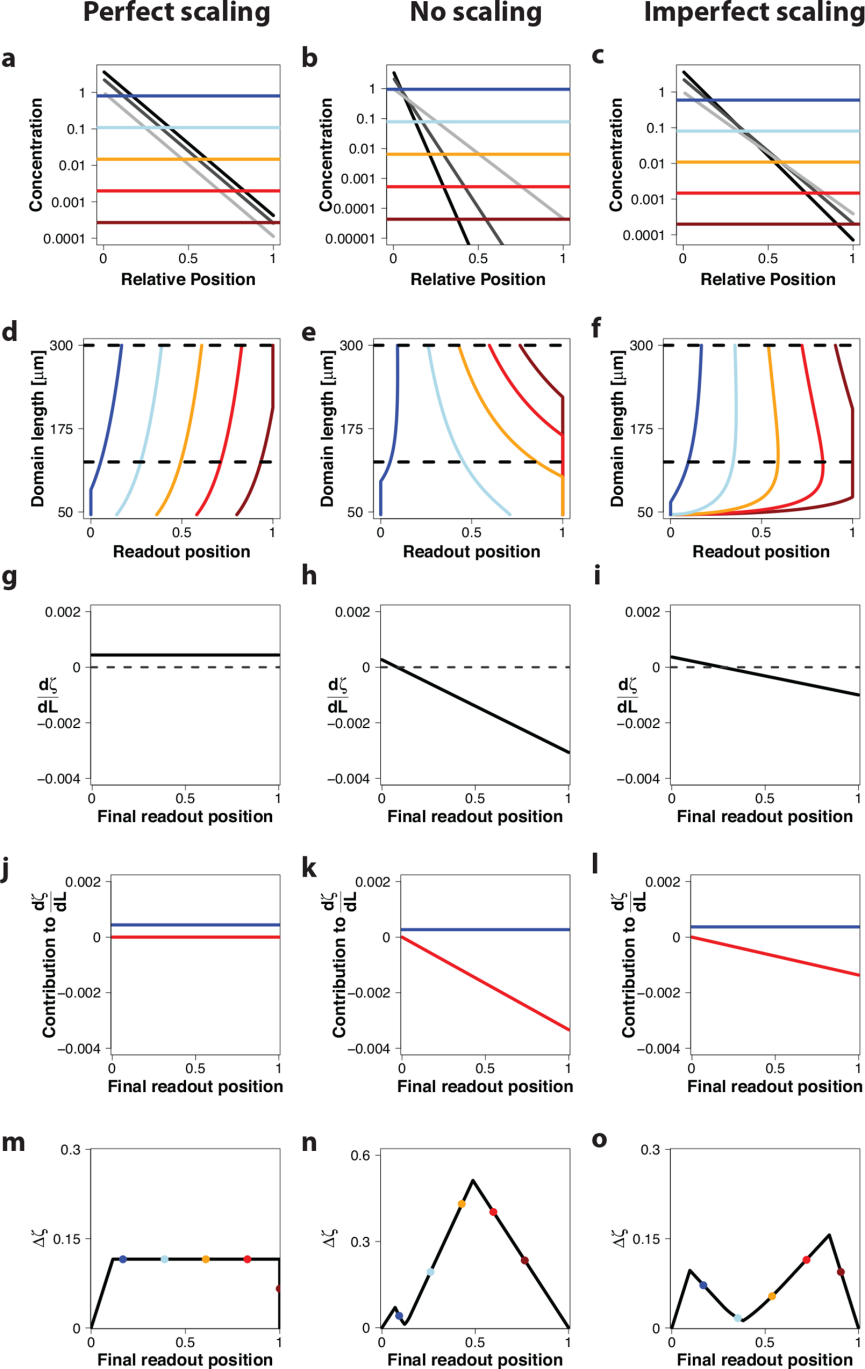
Read-out of morphogen gradients with increasing amplitude *C*_0_ on a growing domain. Gradient read-out for a gradient with an increasing amplitude, *c*_0_ ∼ *L*(*t*)^2*β*^ with *β* = 0.6, in case of (left column) perfect scaling with *λ* = *k*_1_ ⋅ *L*(*t*); *k*_1_ = 0.11 (ref [10]), (center column) absence of scaling with *λ* = 20.2 *μm* (ref [9]), and (right column) imperfect scaling with $\lambda =\sqrt{\frac{D}{2v_{g}}L(t)-\frac{D}{2v_{g}}L_{0}}$; $\frac{D}{2v_{g}}=3\;\mu m$ and *L*_0_ = 46 *μm* (ref [20]). (a-c) A morphogen gradient at three timepoints *L*(*t*) = 100 μm (light grey), *L*(*t*) = 200 μm (dark grey), *L*(*t*) = 300 μm (black)) on a rescaled domain. Differently colored lines represent different concentration thresholds. (d-f) Trajectories of the positions, *ζ*_*θ*_, where the different threshold concentration, shown in panels a-c, are attained. The dashed horizontal lines mark the time interval that is further analysed in panels m-o. (g-i) The derivative of the relative read-out position with respect to the domain length, $\frac{d\zeta_{\theta }}{dL}$. (j-l) The derivative $\frac{d\zeta_{\theta }}{dL}$ not only depends on the relative position *ζ*_*θ*_ (red lines), but also on the gradient amplitude, *β* (blue lines); see Methods section. (m-o) The maximal deviation of the relative read-out positions, Δ*ζ*, in the interval from 125 μm to 300 μm (marked by dashed horizontal lines in panels d-f) for different final read-out positions. The colored dots indicate the final read-out position of the thresholds shown in panels a-c.

Before we focus on the detailed model of the Dpp morphogen in the *Drosophila* wing disc, we will first use a simple set-up to show that the combination of an expanding gradient and an increasing amplitude, as observed for the pre-steady state dispersal mechanism, results in a position on the domain where the concentration remains constant over time. This position can then serve as read-out position. We will consider the simple gradient given by Eq 1, and we will compare the read-out of morphogen gradients that either scale perfectly, i.e. *λ* ∼ *L*(*t*) (Figs 2 and 3, left column), that do not scale at all (*λ* = *const*.) (Figs 2 and 3, center column), or that scale imperfectly as observed in our pre-steady state model of the Dpp gradient ($\lambda ∼\sqrt{t}$) (Figs 2 and 3 right column). By applying different thresholds to the simulated gradients (Figs 2 and 3, differently coloured lines in first row), we can follow the relative read-out position over time on the linearly growing domain, *L*(*t*) = *L*(0) + *v*_*g*_ ⋅ *t* (Figs 2 and 3, second row). Note that the different domain lengths correspond to different time points.

We will first consider the case of a constant amplitude, *c*_0_ = *const*, for the gradient (Fig 2). If the morphogen gradient scales perfectly, i.e. *λ* ∼ *L*(*t*), then the position of the read-out domain, *x*_*θ*_ (Eq 2), will shift proportionally to the increase in the domain length (Fig 2A, differently coloured lines), and the relative read-out position, *ζ*_*θ*_(*t*) (Eq 3), is constant over time (i.e. for all domain lengths, *L*(*t*)) and for all possible concentration thresholds and thus for all possible final read-out positions (Fig 2D, coloured lines). In more mathematical terms, the change of the relative read-out position, *ζ*_*θ*_(*t*), with domain length, *L*(*t*), determined as the derivative, $\frac{d\zeta }{dL(t)}$, from Eq 3 is zero for all times and for all read-out positions (Fig 2G); for a derivation see the Methods section. To evaluate the quality of the read-out on the growing domain we introduce the measure Δ*ζ* (Fig 2J). Δ*ζ* quantifies the maximal shift in the relative read-out position over a time interval Δ*t* (marked by horizontal black lines in panel d for a given threshold concentration *θ* and thus for the different final read-out positions (marked by the different colours in panels a,d,j). Since *ζ*_*θ*_(*t*) does not change over time (Fig 2D and 2G) the maximal time-dependent change in the read-out position, Δ*ζ*, in the considered time interval is also zero for all possible concentration thresholds *θ* and thus for all possible final read-out positions (Fig 2J).

If we now consider a gradient that does not scale at all, i.e. *λ* = *const* (Fig 2B), the relative read-out position is monotonically decreasing with increasing domain length, except at *ζ*_*θ*_(*t*) = 0 (Fig 2E), and $\frac{d\zeta }{dL(t)}$ is therefore negative everywhere, except at *ζ*_*θ*_(*t*) = 0 where it is zero (Fig 2H). Accordingly, Δ*ζ* is nonzero everywhere except for the final boundary positions of zero and one (Fig 2K).

In case of our imperfect scaling mechanism ($\lambda ∼\sqrt{t}$) the relative boundary position is slowly decreasing after a rapid initial increase (Fig 2C). In agreement with this, the deviation in the boundary positions over time is lower than in the absence of scaling (Fig 2F) and while $\frac{d\zeta }{dL(t)}$ is negative everywhere, except at *ζ*_*θ*_(*t*) = 0, the absolute value is smaller than in the absence of scaling (compare Fig 2H and 2I). Accordingly, Δ*ζ* has a similar shape as in the absence of scaling, but the positional inaccuracy is less (compare Fig 2K and 2L). The most stable read-out position is, however, obtained with perfect scaling (Fig 2J).

The situation changes when we consider an increasing gradient amplitude (Fig 3). According to the measurements in the *Drosophila* wing imaginal disc [10], *c*_0_ is related to the area of the wing disc by a power-law of the form

$$c_{0}(t)=A{\left(t\right)}^{\beta }=k_{2}\cdot L{\left(t\right)}^{2\beta }.$$ (4)

Here, *A*(*t*) denotes the area of the wing disc, which is proportional to the square of the length of the domain, *L*(*t*), and *k*_2_ is the proportionality constant.

In case of perfect scaling of the morphogen gradient, *λ*(*t*) ∼ *L*(*t*), we now observe a shift to the right in the read-out position over time (Fig 3A and 3D). From the derivative $\frac{d\zeta }{dL(t)}=\frac{2\beta }{L(t)}k_{1}$ (Fig 3G), we see that this shift depends on the strength, *β*, by which the gradient amplitude, *c*_0_, increases (Fig 3J, blue line), but it does not depend on the threshold concentration and thus on the read-out position (Fig 3J, red line). Accordingly, Δ*ζ* is zero only at the left and right boundaries, and substantially deviates from zero otherwise (Fig 3M).

In case of a non-scaling morphogen gradient, the read-out position still shifts over time (Fig 3B), but the read-out positions close to zero are now shifting to the right rather than to the left (Fig 3E). This effect can be understood by considering the derivative $\frac{d\zeta_{\theta }}{dL}=\frac{2\beta }{L(t)}\frac{\lambda }{L(t)}-\frac{\zeta_{\theta }}{L(t)}$ (Fig 3H). The second term, shown as red line in Fig 3K, is the same as for a constant gradient amplitude (*β* = 0) (Fig 2H). However, as a result of the increasing gradient amplitude (*β* > 0), the derivative now also comprises a term that is independent of the read-out position (Fig 3K, blue line). This additional positive term increases the value for the derivative, such that it is positive for small *ζ*_*θ*_(*t*) and negative for larger *ζ*_*θ*_(*t*) (Fig 3H). Accordingly, close to the source at *ζ*_*θ*_(*t*) ∼ 0.05, there is a position, where the derivative, $\frac{d\zeta_{\theta }}{dL}$, and thus the positional inaccuracy, Δ*ζ*, are zero (Fig 3N). This is visible as a crossing of all gradients over time in a single point (Fig 3B). Such a behavior has indeed recently been reported for the Bicoid gradient in differently sized embryos [24]. In case of the Bicoid gradient, the domains are static but can vary in size in different embryos. DNA replication is coupled to the nuclear volume of the nurse cells such that the concentration is higher in larger embryos (*β* ≈ 3) [24]. In case of the Dpp gradient, the concentration increases less as the domain expands (*β* = 1.2), and as a result the gradients would cross rather close to the source (*ζ*_*θ*_(*t*) ∼ 0.05) in the absence of scaling. Such a short read-out distance would not be helpful for a biological patterning mechanism. Further away from the source the positional inaccuracy, Δ*ζ*, is large again (Fig 3N).

Strikingly, in case of the imperfectly scaling gradient, the read-out position shifts much less over time (Fig 3F), and we again observe a position where all gradients intersect (Fig 3C). Again, the increasing gradient amplitude (*β* > 0) adds a threshold-independent term to the derivative, $\frac{d\zeta_{\theta }}{dL}=\frac{2\beta }{L(t)}\frac{1}{L(t)}\sqrt{\frac{D}{2v_{g}}(L(t)-L_{0})}-\frac{\zeta_{\theta }}{L(t)}+\frac{\zeta_{\theta }}{2\left(L(t)-L_{0}\right)}$. The contribution of this position-independent term is larger than in the absence of scaling (Fig 3K and 3L, blue lines) and the slope of the position-dependent term is less (Fig 3K and 3L, red lines).

As a result, the position *ζ*_*θ*_(*t*), where the derivative is zero (Fig 3I) and the read-out position is thus stable with time, is shifted further into the domain. We note that the derivative, $\frac{d\zeta_{\theta }}{dL}$, in panel i is plotted at the final time point. Here the minimal deviation is attained at *ζ*_*θ*_(*t*) ∼ 0.3. Integrated over time, Δ*ζ*, however, attains the lowest value close to 40% of relative domain length (Fig 3O).

In summary, perfect scaling of morphogen gradients results in a perfectly stable read-out position in case of a constant gradient amplitude, while imperfect scaling gradients result in the most stable read-out position of all three mechanisms in case of increasing gradient amplitudes.

### Read-out of the Dpp gradient in the Drosophila wing disc

**Fig 4:**
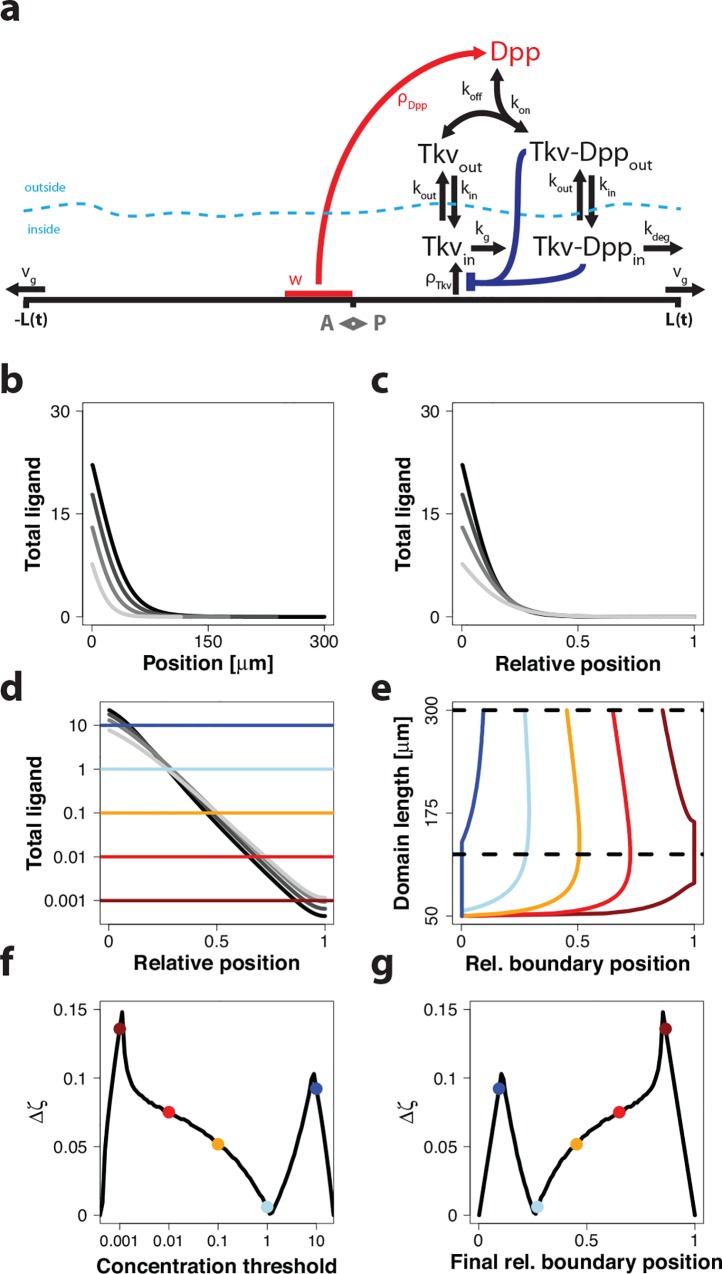
Read out of the Dpp gradient in the *Drosophila* wing disc. (a) A cartoon of the model for Dpp spreading in the wing disc. For details see main text and Methods. (b, c) The simulated gradient profiles of total Dpp both increase in amplitude and expand with the growing domain as shown (b) on the absolute domain, and (c) on a rescaled domain. Time points shown (light to dark grey): 24 h, 46 h, 68 h, 90 h. (d) Total ligand concentration of the simulated profiles shown on a logarithmic scale. Different colors represent different concentration thresholds. (e) Depending on the concentration thresholds, the read-out positions follow different trajectories as the domain length expands over time. The dashed, black horizontal lines indicate the domain length interval used to calculate the positional inaccuracy in the read-out position, Δ*ζ*, shown in panels f,g. (f) The inaccuracy in the read-out position, Δ*ζ*, for different concentration thresholds, plotted on a logarithmic scale. (g) The inaccuracy in the read-out position, Δ*ζ*, has a minimum at around 25% of the domain length. A rather broad range of possible final boundary positions exhibits small positional inaccuracies. Panels a and b were reprinted from [20] under a CC BY license, with permission from Nature Publishing Group & Palgrave Macmillan, original copyright 2014.

We recently developed a model for Dpp spreading in the growing *Drosophila* wing imaginal disc (Fig 4A) that quantitatively agrees with the measured experimental data[20]. In particular, the model reproduces the experimentally observed scaling of the morphogen gradient with domain size, the increase of the gradient amplitude, and the relative distributions of the ligand Dpp between the extracellular and intracellular space. The model follows the dynamics of the morphogen Dpp and its receptor Tkv and considers the internalization and degradation of the Dpp-Tkv complex as two separate steps (Fig 4A). Importantly, degradation of the internalized Dpp ligand is slow, thus ensuring that the model remains in a pre-steady state over the course of the developmental period of 90 hours. The ligand Dpp is produced in a stripe in the center of the domain that spans 10% of the entire anterior-posterior length. The receptor can be produced everywhere, but receptor production is inhibited by ligand-receptor signaling [25,26]. The domain comprises the entire anterior-posterior axis with *x*(*t*) ∈ [−*L*(*t*),*L*(*t*)] and expands linearly at the measured speed *v*_*g*_, i.e. *L*(*t*) = *L*(0) + *v*_*g*_ ⋅ *t*; for more model details, see the Methods section.

When we simulate the model on the growing domain (Fig 4B), we notice that the morphogen gradients at different time points all cross at a relative position of approximately *ζ* = 0.25 (Fig 4C and 4D), even though the gradients widen over time to achieve (imperfect) scaling (Fig 4B). We next monitored the change in the read-out position over time for different threshold concentrations (Fig 4D, differently coloured lines). During the early phase of growth when scaling is particularly imperfect, the relative read-out position, *ζ*_*θ*_(*t*), where a given threshold concentration is attained, is pushed away from the source as the domain is growing out. However, after this initial movement of the read-out positions, the relative read-out positions are remarkably stable on the growing domain (Fig 4E). Much as in Figs 2 and 3, we next plotted the maximal deviations in the read-out position in the time interval that is marked by black horizontal lines in panel e for the different threshold concentrations (Fig 4F) and thus at the different possible final relative read-out positions (Fig 4G). At a threshold concentration of 1 (marked by a light blue line), the read-out position barely changes over time (Fig 4D–4F). Accordingly, at about 25% of domain length, the read-out position in the domain barely changes over time (Fig 4G), even though the amplitude of the gradient increases over time (Fig 4B). We note that the position of this stable read-out position is closer to the source for the detailed Dpp model (25% of domain length) compared to the simple model in Fig 3 (40% of domain length). This difference can be accounted to the impact of ligand degradation. In Figs 2 and 3, we considered the case $\lambda ∼\sqrt{t}$. This relationship is accurate only in the complete absence of degradation[20].

**Fig 5:**
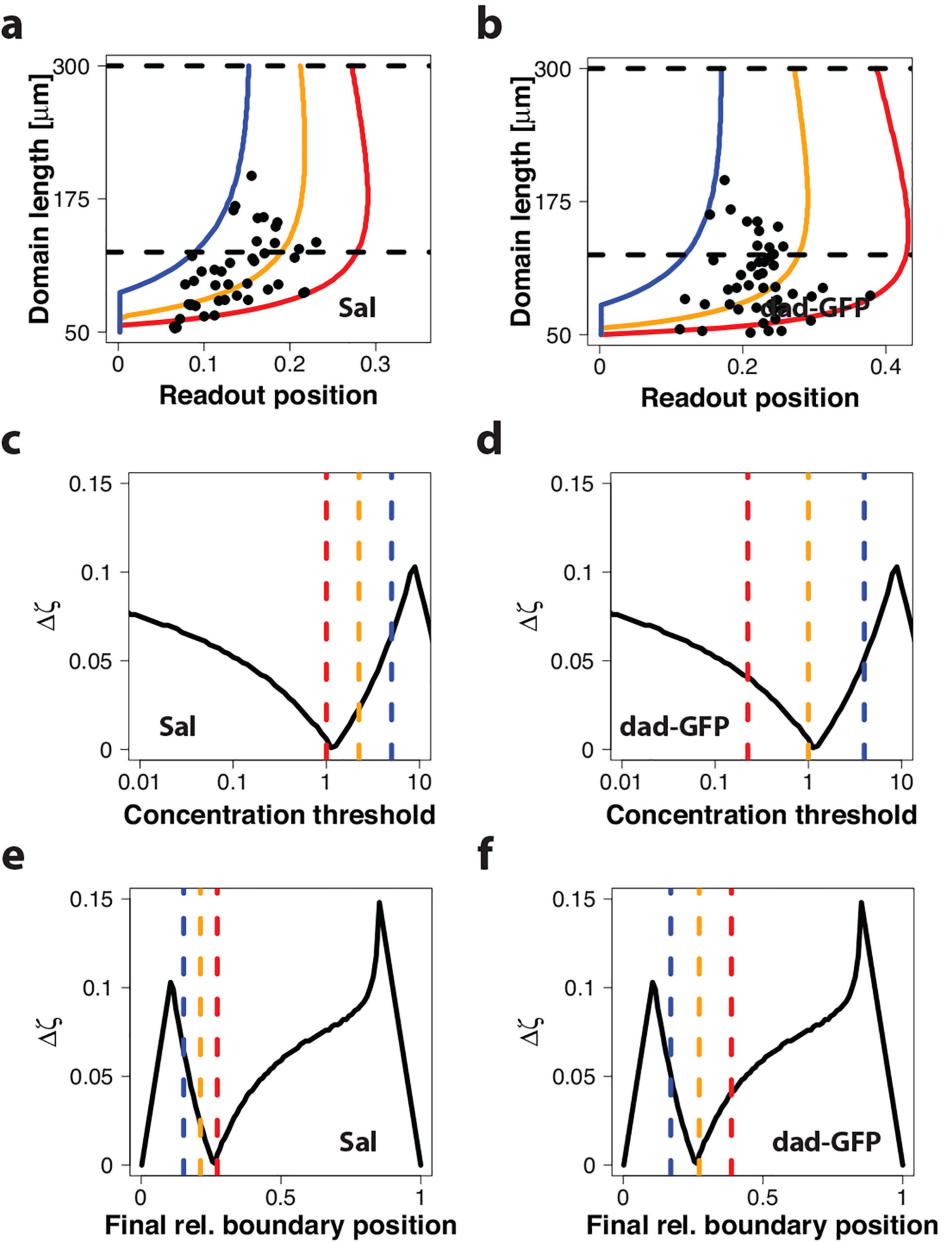
Comparison of predicted and measured expression boundaries of Dpp-controlled genes in the *Drosophila* wing disc. (a, b) Measured relative boundary position over the posterior compartment length for (a) *sal* and (b) *dad-gfp* are shown as black dots [21] and are compared to simulation results for three different thresholds. The thresholds were set to cover the extreme data points (red and blue lines), as well as the average of the data (orange line). (c-f) The maximal deviation in the relative read-out position in the interval from 125 μm to 300 μm (marked by dashed, black lines in a,b), Δ*ζ*, for (left column) *sal* and (right column) *dad-gfp* for (c,d) different threshold concentrations, and (e,f) different relative boundary positions. Vertical coloured lines represent the final positions of the different trajectories that encapsulate the data in (a, b). The range is narrower for *sal* than for *dad-gfp*.

In the *Drosophila* wing disc, several genes are directly regulated by Dpp signaling, in particular *sal* and *dad* [11,12,21]. Their gene expression boundary positions have been reported at different developmental stages, and thus for different wing disc lengths [21]. When we plot the relative boundary positions of these gene expression domains against the domain length we notice a considerable variance in the reported results, both for *sal* (Fig 5A) and for *dad-gfp* (Fig 5B). We can use our model to determine the range of threshold concentrations that would correspond to the extreme measurements (blue and red lines) and to the average measurements (yellow lines). We notice that the experimentally observed positional inaccuracy corresponds to a five-fold variation in the threshold concentration for *sal* and to an 18-fold range for *dad-gfp* (Fig 5C and 5D). When we compare the corresponding relative final read-out positions to the model, we notice that the average biological read-out position (orange line) coincides with the position where the model predicts the highest stability of the read-out position over time (Fig 5C and 5D). In particular, the expression domain of *dad-gfp* coincides almost perfectly with the minimum (Fig 5D), while the *sal* expression boundary is centered at a slightly higher threshold concentration where the read-out position varies slightly more over time (Fig 5C). In conclusion, *sal* and *dad* have their expression boundaries in a part of the domain where the imperfect scaling of the morphogen gradient is compensated by the increasing gradient amplitude, *c*_0_. This provides an explanation how a simple Dpp concentration threshold-based mechanism can yield the observed scaled expression boundaries for these genes.

In contrast to this it is well known that other genes affected by Dpp signaling, e.g. *omb* and *brk*, have their gene expression boundary in more lateral regions of the posterior compartment, but nevertheless have been reported to scale [21]. In these regions our model however would predict that the scaling quality is rather bad (Δ*ζ* ≥ 0.05). The observed lateral scaling behavior can thus not be explained with the read-out of a constant threshold of an imperfectly scaling Dpp gradient. There are two alternative explanations: either the Dpp gradient is read out by a different mechanism in the lateral region, or the scaling of the lateral gene expression domains is achieved by a different morphogen. With regard to the first option, we note that the Dpp levels are at least 2–3 orders of magnitude smaller in lateral regions compared to the maximal concentration at the source. Given the low Dpp concentration in the lateral region, a robust read-out the Dpp concentration can be considered difficult also with a different mechanism. With regard to the second option of a different morphogen, there is a very good candidate, the BMP ligand Glass bottom-boat (Gbb). Much as Dpp signaling, also Gbb signaling is transduced via Tkv type I receptors and pMad [23]. Gbb mutants show defects or complete loss of the L5 vein, which is well established to be controlled by Brk and Omb [27]. Reducing *dpp* function, on the other hand, clearly affects pattern in medial regions (loss of L2 and L4 in strong phenotypes), but never leads to a loss of the L5 vein [23]. These results suggest that in wildtype discs Dpp signaling is the major regulator in medial parts and Gbb signaling is the major regulator in lateral regions. Overexpression of Dpp in the lateral parts will, of course, still affect *brk* and *omb* expression via Dpp’s effects on pMad.

### Influence of parameters on read-out dynamics

**Fig 6:**
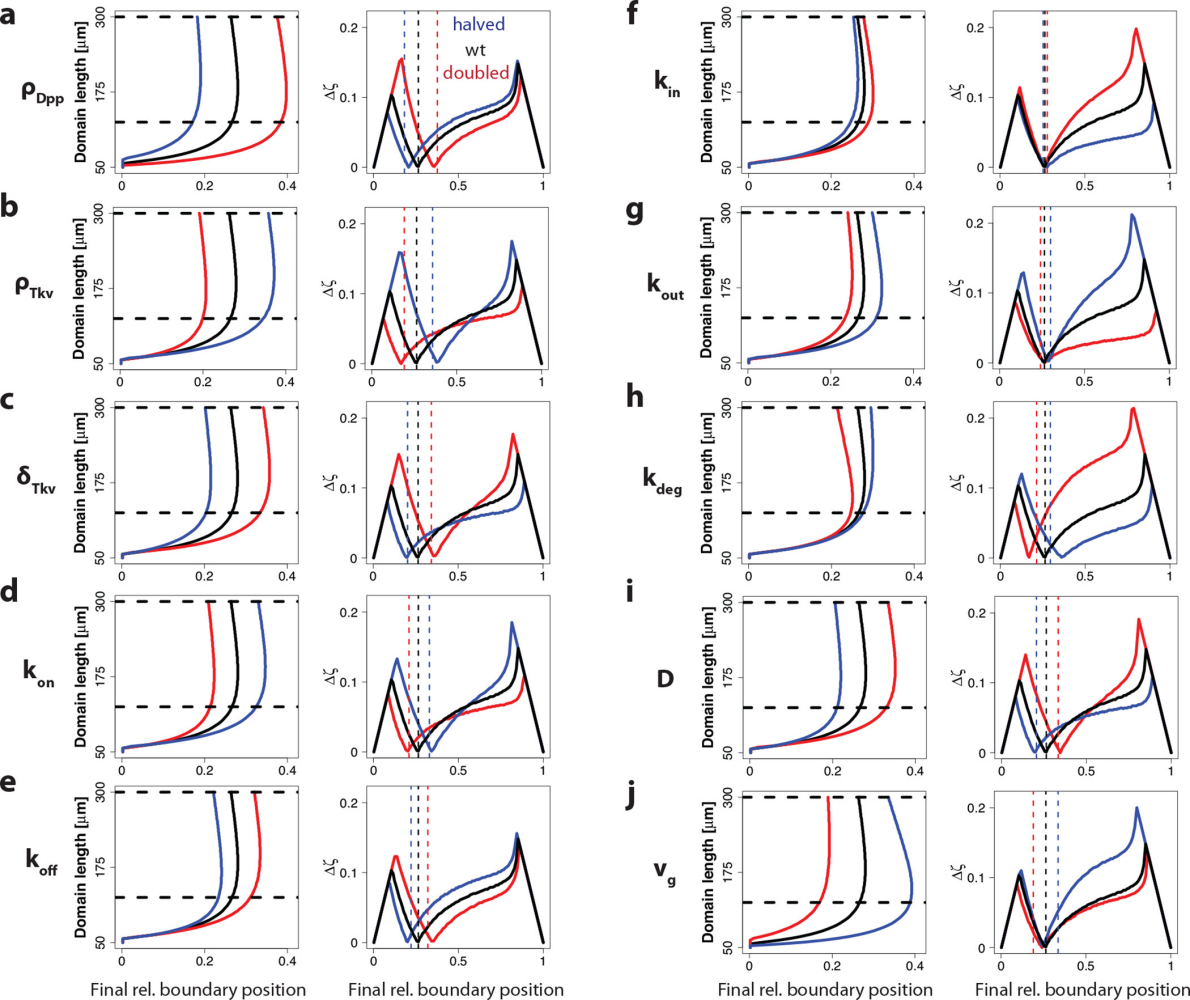
The sensitivity and robustness of the read-out to independent changes in the parameter values. Influence of either doubling (red) or halving (blue) the specified model parameters of the model shown in Fig 4 (left column) on the relative boundary position during domain growth, and (right column) on the maximal deviation in the relative read-out position in the interval from 125 μm to 300 μm (marked by horizontal dashed black lines in a,b), Δ*ζ*, for the different final read-out positions. The perturbed parameters were the (a) Dpp production rate, (b) Tkv production rate, (c) Tkv degradation rate, (d) Dpp-Tkv binding rate, (e) Dpp-Tkv unbinding rate, (f) Tkv internalization rate, (g) Tkv exocytosis rate, (h) degradation rate of internalized Dpp, (i) Dpp diffusion constant, (j) growth speed. Dashed lines indicate the final readout position after doubling (red) or halving (blue) the original parameter value (final point in the panels in the left column). In all perturbations, the concentration threshold was applied which yields the lowest positional inaccuracy in the standard model, *θ* = 1.12 (Fig 4F).

Developmental patterning mechanisms are intriguingly robust, and the correct relative patterns are observed in embryos that differ in size and growth speed or that grow up at different temperatures [28–30].We wondered how robust our conclusions would be to changes in the parameter values. To this end, we both doubled and halved the original value for each of the ten parameters. As a concentration threshold we used the concentration that results in a minimal shift of the relative read-out position for the original parameters. As expected, for most parameters, halving or doubling the parameter values had an effect on the final read-out position (Fig 6, left column). Interestingly, as the read-out position shifted, so did the position, where the most stable read-out is achieved. As a consequence, the most stable read-out was still achieved at the same threshold concentration, even though the position of its read-out had shifted in the domain (Fig 6, right column). One exception to this is the degradation rate *k*_deg_, for which we observe a much higher deviation in the relative boundary position during growth when the degradation rate is increased (Fig 6H). This can be accounted to the lower quality of scaling because a steady state is attained more quickly for a higher degradation rate [20]. As a result, the expansion of the gradient and the increase of *c*_0_ are not compensating each other very well at higher degradation rates. This might also explain the effects seen in Pentagone (pent) mutants, where the pMad gradient is much shorter and most target genes are not scaling [21]. How Pentagone is regulating Dpp is not known yet, and a number of mechanisms have been suggested, including blocking rapid internalization of Dpp, preventing degradation of external Dpp and hindering tight binding of Dpp to Tkv [31]. As discussed above an increase in the rate of degradation, whether mediated by rapid internalization of the Tkv-Dpp complex or the external degradation of Dpp, would reduce scaling and shorten the Dpp gradient. Furthermore there is a strong effect on the size and shape of the wing disc in Pentagone mutants [21,31]. We previously showed that changes in the growth rate or in the linearity of growth can have severe effects on scaling, which would again be in agreement with the qualitative observations of the Pentagone mutant.

### Dual effect of Dpp on growth and patterning enhances robustness of the read-out mechanism to changes in the Dpp production rate

**Fig 7:**
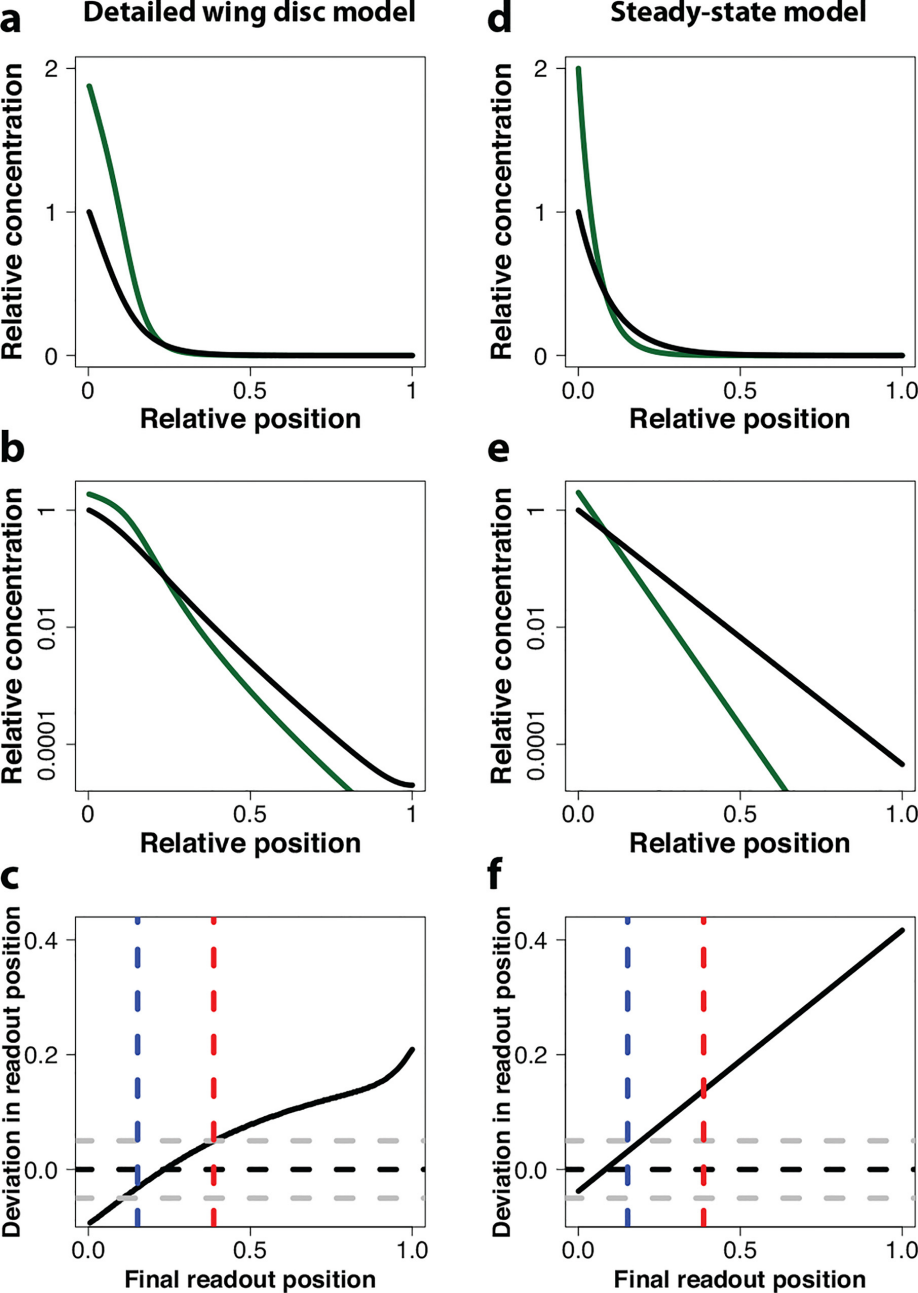
The read-out position is robust to parallel changes in the Dpp production rate and the growth rate in the pre-steady state wing disc model, but not in the steady-state model. (a-b) Impact of a 2-fold increase in the Dpp production rate and the growth rate (green line) on the Dpp gradient compared to the standard parameterization (black line) in the detailed wing disc model on a linear scale (a) and a log-scale (b). (c) The deviation in the final relative readout position between the black and the red gradient in the detailed wing disc model. Gray dashed lines indicate absolute deviations of 0.05. Colored dashed lines correspond to the predicted upper and lower final readout positions of the data shown in Fig 5. (d,e) Impact of a 2-fold increase in the Dpp production rate and the growth rate (green line) on the Dpp gradient compared to the standard parameterization (black line) in a steady-state model on a linear scale (d) and a log-scale (e). (f) The deviation in the final relative readout position between the black and the red gradient in the steady-state model. Gray dashed lines indicate absolute deviations of 0.05. Colored dashed lines correspond to the predicted upper and lower final readout positions of the data shown in Fig 5.

As shown above, an isolated change in the production rate of Dpp, *ρ*_*Dpp*_, greatly shifts the read-out pattern in our model (Fig 6A). However, even though ectopic expression of Dpp has previously been shown to result in considerable overgrowth of the wing disc [32], the Dpp overexpressing wing discs are considered as patterned normally [10]. Interestingly, when we increase the Dpp production rate, *ρ*_*Dpp*_, and the growth rate, *v*_*g*_, by 2-fold and in parallel, we notice that in the region of *sal* and *dad* expression the effect on the simulated Dpp gradient is small both on a linear scale (Fig 7A) and on a log-scale (Fig 7B). Therefore, also the change in the relative read-out position in this region is very small (Fig 7C).

If we compare this to the situation in a simple steady-state model (Methods), we notice that the insensitivity of the Dpp gradient (Fig 7D and 7E) and the relative read-out position (Fig 7F) to a parallel increase in the Dpp production rate and the growth rate is restricted to the very front of the domain. The region, where *sal* and *dad* have their expression boundaries, therefore still become shifted (Fig 7F). The reason for the different levels of compensation in the steady state model and in the pre-steady state wing disc model lie in the different effects of the enhanced Dpp production rate on the Dpp gradient length. In the steady-state model, the Dpp gradient length remains unchanged. On a faster growing, larger domain, the relative gradient length therefore becomes shorter and less Dpp reaches the read-out positions of *sal* and *dad*. In case of the pre-steady state gradient in the detailed wing disc model, the increased Dpp production rate, not only translates into a higher gradient amplitude *c*_0_, but also into a longer gradient length. In fact, the more Dpp is produced, the more saturated the Dpp receptors become, and this effect is even more enhanced by the negative feedback of Dpp signaling on the receptor production rate. The parallel impact of Dpp on growth and patterning thus permits robust patterning in spite of variations in the strength of Dpp production in the pre-steady state wing disc model.

## Discussion

Patterning during development is remarkably robust, but the mechanistic basis has remained elusive. We have previously shown that the scaling of the Dpp gradient with the expanding size of the wing disc [10] can be explained with the pre-steady state behavior of the gradient [20]. However, how a scaled gradient with an increasing amplitude could be read out by cells remained unclear. We have shown here that the pre-steady state character of the gradient also permits the robust threshold-based read-out of the gradient, even though the gradient amplitude increases continuously with time. In particular, we find that the combination of imperfect scaling and a continuously increasing gradient amplitude results in a position on the scaled domain, where the concentration remains constant over development (Figs 2–4). We further found that the position, where the morphogen concentration is constant over time, coincides with the expression boundaries of the two genes, *sal* and *dad*, that are controlled by Dpp signaling (Fig 5) [21].

The read-out mechanism is remarkably robust. Changes in any of the parameter values shift the final read-out position of the gradient, but a constant relative read-out position over time is still achieved for the same concentration (Fig 6). Thus, even if parameter values change, the larvae can still use the same concentration threshold for the read-out. Alternatively, the expression boundary may be detected as the position where the local concentration no longer increases over time. Thus, after an initial build up of the gradient, the morphogen concentration continues to increase over time at positions closer to the source, but decreases at the positions further away (Fig 4D).

It is a long-standing question why Dpp controls both growth and patterning. We now find that the additional role of Dpp in controlling growth is important to enable the observed robustness of the patterning process to changes in the strength of Dpp expression (Fig 7). If Dpp did not enhance growth, Dpp-dependent patterns would be rather sensitive to changes in the Dpp production rate (Fig 6A). The impact of Dpp on growth may thus mainly exist to enhance the robustness of the patterning mechanism to noisy protein production. It has previously been suggested that the Dpp gradient would be responsible for the the uniform growth rate and its decline in the wing disc because, in case of a perfectly scaling exponential gradient, the relative temporal change in the Dpp concentration would be equal everywhere within the domain, thus enabling uniform growth, and the magnitude of the relative temporal change in the Dpp concentration would decline according to a power law, thus offering an explanation for the uniformly declining growth rate (see Methods section for details) [10]. The mechanism has been questioned because clones in the wing imaginal disc that cannot transmit Dpp signalling grow at the same rate as signalling competent cells [33]. In light of our analysis, the Dpp gradient appears to be in pre-steady state and to scale imperfectly. The relative temporal change in the Dpp concentration would then be different at different positions within the domain (S1A Fig), as would be its temporal decline (S1B Fig). Our imperfectly scaling gradient would thus not correlate with the observed growth pattern in the wing disc and our analysis would thus not support such a role of the Dpp gradient.

In summary, our analysis shows that a pre-steady state gradient model enables a robust threshold-based read-out on a growing domain, in spite of a continuously increasing amplitude. Pre-steady-state dynamics are pervasive in morphogen-controlled systems, thus making this a probable general mechanism for the scaled read-out of morphogen gradients in growing developmental systems.

## Methods

In this paper, two models are used. In Figs 2 and 3 we use the observation that the experimentally measured Dpp gradient can be well approximated by exponential gradients [10]. We then analyze three different modes of scaling for a constant gradient amplitude (Fig 2) as well as an increasing gradient amplitude (Fig 3). In the absence of scaling the characteristic length *λ* is constant over time. In case of perfect scaling the characteristic length is proportional to the domain length, i.e. *λ* ∼ *L*(*t*). We previously showed that the scaling of the Dpp gradient can be best described by the pre-steady state dispersal of a diffusive, long-lived morphogen [20]. We showed that for such a behavior the characteristic length can be approximated by $\lambda ∼\sqrt{t}$, which we termed imperfect scaling. The analysis shown in Figs 2 and 3 is thus based on the biologically observed exponential gradient approximation and not on any steady-state solution of a mechanistic model although it converges to the same mathematical description in the case of a constant gradient amplitude and a constant gradient length. In Figs 4–6 and 7A–7C, we confirm the validity of the results from the simple models (Figs 2 and 3) in a detailed pre-steady state model of wing disc patterning. We previously showed that the detailed model recapitulates all relevant experimental measurements quantitatively [20]. In Fig 7D–7F we use the steady-state solution for a secretion-diffusion-decay model, which follows the same mathematical description as Eq 1.

### Derivation of the relative read-out positions over time for the different scaling behaviours

In the following, we provide the derivation of the relative read-out position and the change of the read-out position on the growing domain for the different scaling behaviours, i.e. for perfect scaling, no scaling, and imperfect scaling, when the gradient amplitude is either constant or increases with time.

Eq (3) reports the general formula for the relative read-out position, $\zeta_{\theta }(t)=\frac{\lambda }{L(t)}\log\left(\frac{c_{0}}{\theta }\right)$. This formula applies in the absence of scaling, *λ* = *const*. For perfect scaling we have *λ* = *k*_1_ ⋅ *L*(*t*), while for imperfect scaling, we previously established that *E*[*x* ^2^] = 2*λ* ^2^ = *Dt* (ref [20]) such that $\lambda =\sqrt{\frac{D\cdot L(t)}{2v_{g}}-\frac{D\cdot L_{0}}{2v_{g}}}$ on a linearly growing domain with *L*(*t*) = *L*_0_ + *v*_*g*_ ⋅ *t*.

We can substitute the respective length scales into Eq (3) and obtain the relative read-out position for

$$\text{Perfect scaling}:\;\zeta_{\theta }=\frac{k_{1}\cdot L(t)\cdot \log\left(\frac{c_{0}}{\theta }\right)}{L(t)}=k_{1}\cdot \log\left(\frac{c_{0}}{\theta }\right)$$ (5)

$$\text{Imperfect scaling}:\;\zeta_{\theta }=\frac{\sqrt{\frac{D\cdot L(t)}{2v_{g}}-\frac{D\cdot L_{0}}{2v_{g}}}\cdot \log\left(\frac{c_{0}}{\theta }\right)}{L(t)}.$$ (6)

For all aforementioned cases we can also calculate the derivative of the relative boundary position with respect to the domain length. To obtain this measure we differentiate Eqs (3,5 and 6) w.r.t. the domain length and substitute Eqs (3,5 and 6) into them. The resulting derivatives are:

$$\text{Non-scaling}:\;\frac{d\zeta_{\theta }}{dL}=-\frac{\zeta_{\theta }}{L(t)}$$ (7)

$$\text{Perfect scaling}:\;\frac{d\zeta_{\theta }}{dL}=0$$ (8)

$$\text{Imperfect scaling}:\;\frac{d\zeta_{\theta }}{dL}=-\frac{\zeta_{\theta }}{L(t)}+\frac{\zeta_{\theta }}{2\cdot \left(L(t)-L_{0}\right)}.$$ (9)

We now consider the impact of an increasing gradient amplitude. According to Eq (4) we have *c*_0_ = *k*_2_ ⋅ *L*(*t*)^2*β*^ and thus, with Eqs (3,5 and 6), we have for the relative read-out position:

$$\text{Non-scaling}:\;\zeta_{\theta }=\frac{\lambda \cdot \log\left(\frac{k_{2}\cdot L{\left(t\right)}^{2\beta }}{\theta }\right)}{L(t)}$$ (10)

$$\text{Perfect scaling}:\;\zeta_{\theta }=k_{1}\cdot \log\left(\frac{k_{2}\cdot L{\left(t\right)}^{2\beta }}{\theta }\right)$$ (11)

$$\text{Imperfect scaling}:\;\zeta_{\theta }=\frac{\sqrt{\frac{D\cdot L(t)}{2v_{g}}-\frac{D\cdot L_{0}}{2v_{g}}}\cdot \log\left(\frac{k_{2}\cdot L{\left(t\right)}^{2\beta }}{\theta }\right)}{L(t)}.$$ (12)

For all cases we can then again calculate the derivative of the relative boundary position with respect to the domain length by differentiating and then substituting Eq 10–12 into them:

$$\text{Non-scaling}:\;\frac{d\zeta_{\theta }}{dL}=-\frac{\zeta_{\theta }}{L(t)}+\frac{2\beta \cdot \lambda }{L{\left(t\right)}^{2}}$$ (13)

$$\text{Perfect scaling}:\;\frac{d\zeta_{\theta }}{dL}=\frac{2\beta \cdot k_{1}}{L(t)}$$ (14)

$$\text{Imperfect scaling}:\;\frac{d\zeta_{\theta }}{dL}=-\frac{\zeta_{\theta }}{L(t)}+\frac{\zeta_{\theta }}{2\left(L(t)-L_{0}\right)}+\frac{2\beta \cdot \sqrt{\frac{D\cdot L(t)}{2v_{g}}-\frac{D\cdot L_{0}}{2v_{g}}}}{L{\left(t\right)}^{2}}.$$ (15)

We note that for all these formulas, we can recover the formulas for constant gradient amplitude by setting *β* = 0.

### Deviation of the final read-out position in the steady-state gradient model

Assuming a steady-state model as given by Eq (1), we can calculate the deviation in the final read-out position between a wildtype length scale *λ*_*wt*_ and a new length scale *λ*_*new*_.

$$\zeta_{wt}-\zeta_{new}=\zeta_{wt}-\frac{\lambda_{new}\cdot \log\left(\frac{C_{0}}{\theta }\right)}{L(t)}=\zeta_{wt}-\frac{\lambda_{new}\cdot \frac{x_{wt}}{\lambda_{wt}}}{L(t)}=\zeta_{wt}-\zeta_{wt}\cdot \frac{\lambda_{new}}{\lambda_{wt}}=\zeta_{wt}\left(1-\frac{\lambda_{new}}{\lambda_{wt}}\right)$$ (16)

Similarly we can also calculate the deviation in the final read-out position for parallel changes in the production rate and the growth speed. In the steady-state model the production rate is proportional to the concentration at the left boundary *c*_0_. We can therefore write

$$\zeta_{wt}-\zeta_{new}=\frac{\lambda \cdot \log\left(\frac{c_{0}^{wt}}{\theta }\right)}{L_{0}+v_{g}^{wt}\cdot t}-\frac{\lambda \cdot \log\left(\frac{c_{0}^{new}}{\theta }\right)}{L_{0}+v_{g}^{new}\cdot t}=\zeta_{wt}-\frac{\lambda \cdot \log\left(f\right)+\zeta_{wt}\cdot \left(L_{0}+v_{g}^{wt}\cdot t\right)}{L_{0}+f\cdot v_{g}^{wt}\cdot t}$$ (17)

where *f* is the factor by which *c*_0_ and *v*_*g*_ are changed, i.e. $v_{g}^{new}=f\cdot v_{g}^{wt}$ and $c_{0}^{new}=f\cdot c_{0}^{wt}$.

### Gradient read-out based on local temporal changes in the morphogen concentration

It has been suggested that, rather than responding to fixed concentration thresholds, cells may sense the relative temporal increase of the concentration over time, i.e. $\frac{dc/dt}{c}=\frac{\dot{c}}{c}$ (ref [10,34]). In the following, we will derive the formulas for the relative temporal increase of the concentration, $\frac{\dot{c}}{c}$, for perfect scaling, absence of scaling, and imperfect scaling. As before, we are considering a morphogen gradient of the form *c*(*x*,*t*) = *k*_2_ ⋅ *L*(*t*)^2*β*^ ⋅ exp(−*x* / *λ*(*t*)) on a linearly growing domain *L*(*t*) = *L*(0) + *v*_*g*_ ⋅ *t*, with *x* = *ζ* ⋅ *L*(*t*).

Assuming perfect scaling, we have *c*(*ζ*,*t*) = *k*_2_ ⋅ (*L*_0_ + *v*_*g*_ ⋅ *t*)^2*β*^ ⋅ exp(−*ζ* / *k*_1_). The temporal derivative is given by $\dot{c}(\zeta ,t)=k_{2}\cdot 2\beta \cdot v_{g}\cdot {\left(L_{0}+v_{g}\cdot t\right)}^{2\beta -1}\cdot \exp(-\zeta /k_{1})$. Therefore the normalized temporal increase of the concentration over time is given by:

$$\frac{\dot{c}(\zeta ,t)}{c(\zeta ,t)}=\frac{k_{2}\cdot 2\beta \cdot v_{g}\cdot {\left(L_{0}+v_{g}\cdot t\right)}^{2\beta -1}\cdot \exp(-\zeta /k_{1})}{k_{2}\cdot {\left(L_{0}+v_{g}\cdot t\right)}^{2\beta }\cdot \exp(-\zeta /k_{1})}=\frac{2\beta \cdot v_{g}}{L_{0}+v_{g}\cdot t}=\frac{2\beta \cdot v_{g}}{L(t)}$$ (18)

Similarly, we can calculate the normalized temporal increase of the concentration over time in the case of absence of scaling:

$$\begin{array}{l} \frac{\dot{c}(\zeta ,t)}{c(\zeta ,t)}=\frac{k_{2}\cdot \exp\left(-\frac{\zeta \cdot L(t)}{\lambda }\right)\cdot \left(2\beta \cdot v_{g}\cdot L{\left(t\right)}^{2\beta -1}-L{\left(t\right)}^{2\beta }\cdot \frac{v_{g}}{\lambda }\cdot \zeta \right)}{k_{2}\cdot L{\left(t\right)}^{2\beta }\cdot \exp\left(-\frac{\zeta \cdot L(t)}{\lambda }\right)}\\ \;=\frac{2\beta \cdot v_{g}-L(t)\cdot \frac{v_{g}}{\lambda }\cdot \zeta }{L(t)}=\frac{2\beta \cdot v_{g}}{L(t)}-\frac{v_{g}\cdot \zeta }{\lambda } \end{array},$$ (19)

and in the case of imperfect scaling:

$$\begin{array}{l} \frac{\dot{c}(\zeta ,t)}{c(\zeta ,t)}=\frac{k_{2}\cdot \exp\left(-\frac{\sqrt{2}\cdot \zeta \cdot L(t)}{\sqrt{Dt}}\right)\cdot \left(2\beta \cdot v_{g}\cdot L{\left(t\right)}^{2\beta -1}+L{\left(t\right)}^{2\beta }\cdot \left(\frac{D\cdot \zeta \cdot L(t)}{\sqrt{2}\cdot {\left(Dt\right)}^{\frac{3}{2}}}-\frac{\sqrt{2}\cdot v_{g}\cdot \zeta }{\sqrt{Dt}}\right)\right)}{k_{2}\cdot L{\left(t\right)}^{2\beta }\cdot \exp\left(-\frac{\sqrt{2}\cdot \zeta \cdot L(t)}{\sqrt{Dt}}\right)}\\ \;=\frac{2\beta \cdot v_{g}}{L(t)}-\frac{D\cdot \zeta \cdot \left(L(t)-2\cdot L_{0}\right)}{\sqrt{2}\cdot {\left(\frac{D}{v_{g}}L(t)-\frac{D}{v_{g}}L_{0}\right)}^{\frac{3}{2}}} \end{array}$$ (20)

We see that for perfect scaling, $\frac{\dot{c}}{c}$ is spatially uniform and decreases during growth with time according to a power law. In the absence of scaling and for imperfect scaling, $\frac{\dot{c}}{c}$ still decreases with time, but not in a spatially uniform manner.

### A model for the Dpp gradient in the Drosophila wing imaginal disc

We use our previously published model for Dpp ligand dynamics in the Drosophila wing imaginal disc. The models considers both the diffusible ligand Dpp and the cell-bound receptor Tkv. In summary, the reaction terms *R*_*i*_ for the components *c*_*i*_ in Fig 4A are given as:

$$\begin{array}{l} \;R_{Dpp}=\rho_{Dpp}\Lambda_{Dpp}-k_{on}c_{Dpp}c_{Tkv_{out}}+k_{off}c_{Dpp-Tkv_{out}}\\ R_{Dpp-Tkv_{out}}=k_{on}c_{Dpp}c_{Tkv_{out}}-k_{off}c_{Dpp-Tkv_{out}}-k_{in}c_{Dpp-Tkv_{out}}+k_{out}c_{Dpp-Tkv_{in}}\\ \;R_{Dpp-Tkv_{in}}=k_{in}c_{Dpp-Tkv_{out}}-k_{out}c_{Dpp-Tkv_{in}}-k_{deg}c_{Dpp-Tkv_{in}}\\ \;R_{Tkv_{out}}=-k_{on}c_{Dpp}c_{Tkv_{out}}+k_{off}c_{Dpp-Tkv_{out}}-k_{in}c_{Tkv_{out}}+k_{out}c_{Tkv_{in}}\\ \;R_{Tkv_{in}}=\rho_{Tkv}(x)•\bar{H}+k_{in}c_{Tkv_{out}}-k_{out}c_{Tkv_{in}}-\delta_{Tkv}c_{Tkv_{in}} \end{array}$$ (21)

with the following additional definitions:

$$\rho_{Tkv}(x)=\{\begin{array}{c} \rho_{Tkv}^{0}\\ 0.5•\rho_{Tkv}^{0} \end{array}\begin{array}{cc} \;if & x\notin \Lambda_{Dpp}\\ \;if & x\in \Lambda_{Dpp} \end{array},$$ (22)

$$\bar{H}=\frac{K^{n}}{K^{n}+{\left(c_{Dpp-Tkv_{in}}+c_{Dpp-Tkv_{out}}\right)}^{n}}\;\text{and}$$ (23)

$$\Lambda_{Dpp}(t)=0.2•L(t).$$ (24)

The model is solved on a linearly growing domain is thus formulated as advection-reaction-dispersion equations for a compound *c*_*i*_ with diffusion coefficient *D*_*i*_, velocity field *u* and reaction terms *R*_*i*_:

$$\partial_{t}c_{i}+\nabla (uc_{i})=D_{i}\nabla^{2}c_{i}+R_{i}$$ (25)

We use zero flux boundary conditions for all components, i.e.

$$\nabla c_{i}=0$$ (26)

As initial conditions we use:

$$\begin{array}{l} \;c_{Dpp}(0)=0\\ c_{Dpp-Tkv_{out}}(0)=0\\ \;c_{Dpp-Tkv_{in}}(0)=0\\ \;c_{Tkv_{out}}(0)=\frac{\rho_{Tkv}•k_{out}}{\delta_{Tkv}•k_{in}}\\ \;c_{Tkv_{in}}(0)=\frac{\rho_{Tkv}}{\delta_{Tkv}} \end{array}$$ (27)

The parameter values used in our standard model are as before [20], i.e. *L*_0_ = 50 *μm*, *v*_*g*_ = 7.7 ⋅ 10^−4^ *μm* ⋅ *s* ^−1^, Δ*t* = 90*h*, *D* = 0.1 *μm* ^2^ ⋅ *s* ^−1^, *k*_deg_ = 4 ⋅ 10^−6^ *s* ^−1^, *δ*_*Tkv*_ = 1 ⋅ 10^−4^ *s* ^−1^, *ρ*_*Dpp*_ = 3 ⋅ 10^−4^ *s* ^−1^, *ρ*_*Tkv*_ = 2 ⋅ 10^−3^ *s* ^−1^, *k*_*on*_ = 2 ⋅ 10^−3^ *s* ^−1^, *k*_*off*_ = 4 ⋅ 10^−4^ *s* ^−1^, *k*_*in*_ = 8 ⋅ 10^−4^ *s* ^−1^, *k*_*out*_ = 1 ⋅ 10^−4^ *s* ^−1^, *K* = 10, *n* = 0.5. Concentrations are dimensionless.

### Software

The equations were solved with finite difference methods (FDM) as implemented in MATLAB on uniform growing domains, and by finite element methods (FEM) as implemented in COMSOL Multiphysics 4.3b on non-uniformly growing domains.

## Supporting Information

S1 Fig The predicted relative change in the Dpp concentration within the domain. (a) The relative change in the Dpp concentration at the different relative positions at four time points (light to dark grey): 24 h, 46 h, 68 h, 90 h. The coloured lines mark four different relative positions: 0.2 (blue), 0.4 (orange), 0.6 (red), 0.8 (brown). (b) The relative change in the Dpp concentration decreases over time, albeit differently at different relative positions (colours as in panel a).
(EPS)
